# Complexity of Multi-Channel Electroencephalogram Signal Analysis in Childhood Absence Epilepsy

**DOI:** 10.1371/journal.pone.0134083

**Published:** 2015-08-05

**Authors:** Wen-Chin Weng, George J. A. Jiang, Chi-Feng Chang, Wen-Yu Lu, Chun-Yen Lin, Wang-Tso Lee, Jiann-Shing Shieh

**Affiliations:** 1 Department of Life Science, National Taiwan University, Taipei, Taiwan; 2 Department of Pediatrics, National Taiwan University Hospital, Taipei, Taiwan; 3 Department of Pediatrics, College of Medicine, National Taiwan University, Taipei, Taiwan; 4 Department of Pediatric Neurology, National Taiwan University Children’s Hospital, Taipei, Taiwan; 5 Department of Mechanical Engineering, Yuan Ze University, Taoyuan, Chung-Li, Taiwan; 6 Graduate Institute of Brain and Mind Sciences, National Taiwan University, Taipei, Taiwan; 7 Center for Dynamical Biomarkers and Translational Medicine, National Central University, Chung-Li, Taiwan; 8 Innovation Center for Big Data and Digital Convergence, Yuan Ze University, Taoyuan, Chung-Li, Taiwan; Radboud University Nijmegen, NETHERLANDS

## Abstract

Absence epilepsy is an important epileptic syndrome in children. Multiscale entropy (MSE), an entropy-based method to measure dynamic complexity at multiple temporal scales, is helpful to disclose the information of brain connectivity. This study investigated the complexity of electroencephalogram (EEG) signals using MSE in children with absence epilepsy. In this research, EEG signals from 19 channels of the entire brain in 21 children aged 5-12 years with absence epilepsy were analyzed. The EEG signals of pre-ictal (before seizure) and ictal states (during seizure) were analyzed by sample entropy (SamEn) and MSE methods. Variations of complexity index (CI), which was calculated from MSE, from the pre-ictal to the ictal states were also analyzed. The entropy values in the pre-ictal state were significantly higher than those in the ictal state. The MSE revealed more differences in analysis compared to the SamEn. The occurrence of absence seizures decreased the CI in all channels. Changes in CI were also significantly greater in the frontal and central parts of the brain, indicating fronto-central cortical involvement of “cortico-thalamo-cortical network” in the occurrence of generalized spike and wave discharges during absence seizures. Moreover, higher sampling frequency was more sensitive in detecting functional changes in the ictal state. There was significantly higher correlation in ictal states in the same patient in different seizures but there were great differences in CI among different patients, indicating that CI changes were consistent in different absence seizures in the same patient but not from patient to patient. This implies that the brain stays in a homogeneous activation state during the absence seizures. In conclusion, MSE analysis is better than SamEn analysis to analyze complexity of EEG, and CI can be used to investigate the functional brain changes during absence seizures.

## Introduction

Absence epilepsy is a common generalized epilepsy in children [1]. Its characteristic features are the abrupt cessation of activities and consciousness impairment, with more or less automatisms [2]. The duration of a typical absence seizure is brief, lasting for seconds, without an aura or post-ictal impairment [2]. Electroencephalogram (EEG) is a non-invasive measurement that provides temporal and spatial information regarding the electrical activity of the brain that has been widely used to detect seizures in patients with epilepsy [3]. Ictal EEG in typical absence seizures is characterized by the sudden onset of 3 Hz generalized spike-wave complexes, while the inter-ictal EEG typically shows a normal background.

Over the past decade, the pathophysiology of absence seizures has been extensively studied in both human and animal models, and various cortical or subcortical activations preceding generalized seizures have been documented [4–8]. Dynamic changes in the temporo-spatial course have been shown in typical absence seizures using simultaneous EEG and functional magnetic resonance imaging (EEG-fMRI) [9–15]. However, the detection of seizure dynamics from pre-ictal to ictal states cannot be reached solely using conventional EEG without advanced analysis.

In recent years, advanced algorithm analysis methods have been developed and proposed to analyze the temporo-spatial evolution of EEG recordings [16, 17]. Among these, entropy-based approaches that can characterize the rate of creation of information in dynamic systems have been used for quantifying the “complexity” and “irregularity” of EEG recordings. These approaches may play a leading role in the analysis of biological signals [18–20]. Novel, non-linear entropy measurement methods such as approximate entropy (ApEn), sample entropy (SamEn), and Lempel-Ziv entropy offer the potential to find specific patterns and examine irregularities in a time series [18]. These have been considered to be practical entropy-based measurements in previous researches [21, 22]. However, traditional entropy-based algorithms (e.g., ApEn and SamEn) only quantify the regularity of a time series. A higher entropy value may reflect an increase in the degree of irregularity and randomness but is not guaranteed to have an increase in the complexity of the time series. For example, white noise series have high entropy in scale 1, but this value will decrease quickly when increasing the scales. This means that white noise series have a high degree of irregularity and randomness but low complexity [23,24]. Therefore, in 2002, a multiscale entropy (MSE) analysis that calculates SamEn for each scale over multiple time scales, which defined the complexity index (CI) by calculating the area under the MSE curve to provide a more meaningful measure of complexity, was proposed by Costa et al [23]. At present, MSE and CI have been successfully utilized to analyze several biological signals (e.g., EEG signals) and distinguish healthy status from pathological conditions, including Alzheimer's disease, autism, and epilepsy [23, 24]. More recently, in 2011, Ahmed and Mandic proposed a multivariate MSE (MMSE) algorithm, which can be used to analyze the situation and relationship of each channel [25]. This algorithm can not only investigate the physiological conditions but also investigate the relationship between channel and channel. However, in this study, we applied multiple-channel MSE by computing MSE repeatedly for each channel separately [23]. This is different from multivariate MSE [25]. Ordinary computer speed and memory are not enough for running 19 channels together using MMSE. Because we should clarify the function in the different parts of brain and define the physiological meanings before we apply this algorithm, in this study, we only investigated the difference in MSE in different channels of different patients.

Based on the evidence from these studies as well as our own previous studies, the present study aimed to investigate the role of MSE of EEG signals in children with absence seizures and clarify the variations of CI in their pre-ictal and ictal states. Because the abnormality in “cortico-thalamo-cortical network” may lead to the development of absence seizures [5–7], we wanted to investigate whether the involvement of cerebral cortex can be demonstrated by the changes of MSE and CI in EEG.

## Materials and Methods

### Ethics statement

The Institutional Review Board of National Taiwan University Hospital approved the present study and waived the need for written informed consent from the participants, as the data were analyzed anonymously.

### EEG recording and processing

Data of EEG signals were collected from subjects receiving routine EEG examination. EEG were performed with 19 Ag/AgCl electrodes placed on the scalp at Fp1, Fp2, Fz, F3, F4, F7, F8, Cz, C3, C4, Pz, P3, P4, T3, T4, T5, T6, O1, and O2 electrode sites, following the 10/20 international electrode placement [26]. Thirty-four EEG events were recorded by the digital EEG system (Nicolet) with a sampling frequency of 200 Hz, and 21 EEG events were recorded by the digital EEG system (Nihon Kohden) with a sampling frequency of 1000 Hz using a 16-bit analogue-to-digital converter and filter within a frequency band of 0.5–70 Hz to remove the artifacts. EEG of the patients was performed by sampling frequency of both 1000 Hz and 200 Hz due to the recent installation of a newer machine. The patients’ EEG signals were recorded for 1–2 h continuously to ensure the detection of seizure onset.

The timing of onset and offset of seizure attacks was identified by experienced pediatric neurologists who assessed characteristic clinical manifestations and typical 3–4 Hz generalized symmetric spike-wave discharges lasting >1.0 s. In this research, 15–20 s of EEG data using monopolar montage with average reference in pre-ictal and ictal states were analyzed. Only segments free of artifacts such as eye movements, blinks, muscle movements, or other artifacts were visually identified and selected for analysis. Two sampling frequencies, 200 Hz and 1000 Hz, were used for analysis. The offline EEG data points were extracted for analysis.

To investigate the dynamic changes of EEG signals during different seizure phases, the EEG signals without artifacts were selected and segmented into pre-ictal (10 s before seizure onset) and ictal (intervals during onset and offset of seizure attacks) phases. The ictal phase last for 9–12 s.

### Subjects

The EEG recordings were obtained from 21 children with absence epilepsy, aged 5–12 years. The children were newly diagnosed to have absence epilepsy and were treatment naïve. Among them, 13 (34 seizure episodes) were assessed using the digital EEG system (Nicolet) with a sampling frequency of 200 Hz, while eight (21 seizure episodes) were evaluated using the digital EEG system (Nihon Kohden) with a sampling frequency of 1000 Hz. The demographic data of these children were collected from the Department of Pediatrics, National Taiwan University Hospital.

### Multiscale entropy

**Fig 1:**
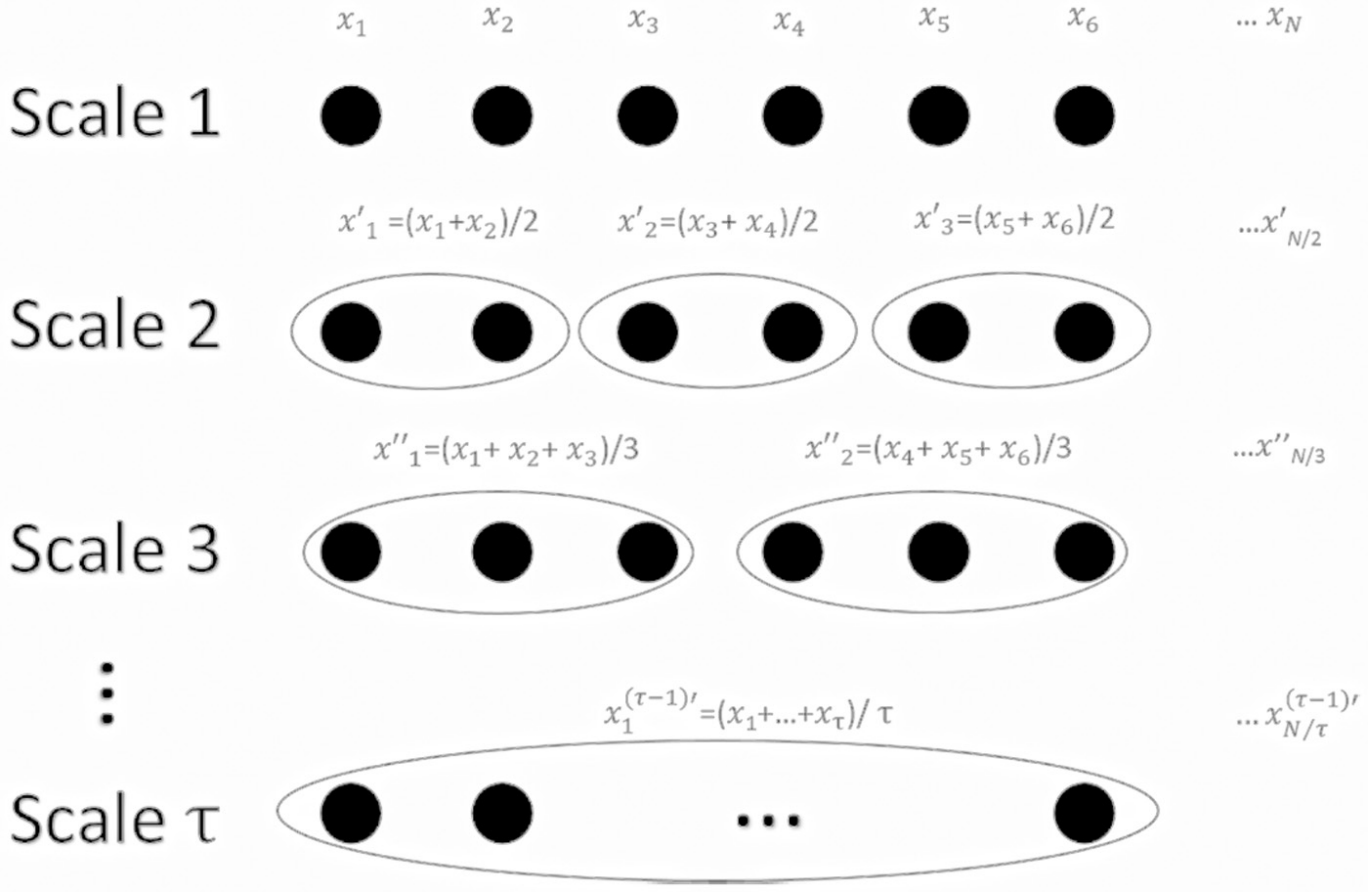
The illustration of the coarse graining procedure for scales 1 to τ.

Entropy was regarded as the index of the degree of randomness of data points. In the MSE analysis, the original EEG time series {*x*_1_,*x*_2_,…,*x*_*N*_} was first coarse-grained by the scale factor (SF) τ to get different new EEG time series (Fig 1). The EEG time series $\left\{y_{i}^{\left(\tau \right)}\right\}$ in different scales could be calculated as follows:

yi(τ)=1τ∑jτi=(j−1)τ+1xi, 1≤j≤Nτ

Entropy was then calculated in different time series. SamEn is a measurement of the irregularity of data points. The calculations of SamEn were expressed as follows:

$$\text{SamEn}(\text{m,r,N})=-\text{ln}\frac{C^{\left(m+1\right)}(r)}{C^{m}(\text{r})}$$

Where

$$C^{m}\left(r\right)={\left(N-m\right)}^{-1}\sum_{}^{}\begin{array}{l} N-m\\ i \end{array}C_{i}^{m}\left(r\right)$$

Therefore, $C_{i}^{m}(r)$ is the number of all probable pairs (*i*,*j*) with *d*<*R* Therein, $d\;=\;\left|x_{i}^{m}-x_{j}^{m}\right|$ denoted the distance between points $x_{i}^{m}$ and $x_{j}^{m}$ in the space of dimension *m*, r is a coefficient of tolerance, SD is a standard deviation of original data, *R* represents the maximum tolerable distance, and *N* is the length of the time series. Various theoretical and clinical applications had proven that SamEn had better statistical validity for *m* = 1 or 2 and 0.1≤*r*≤0.25 [24]. In the present study, *m* = 2, *r* = 0.15, *R* = *r* *SD were applied. Therefore, the value of SamEn could be 0–2.5 according to a previous study using 1000 random data points. The 2.5 indicates the data like random number, and zero indicates that the data are predictable [18]. Furthermore, the scale was set to 20, as in our previous study. Entropy was then calculated for each coarse-grained time series and plotted as a function of the scale factor to get MSE. In the analysis of MSE, SamEn is the entropy in scale 1.

Unlike SamEn, MSE observed the difference on different scales that represented diverse frequencies [22,23]. The analysis of SamEn requires enough data points to calculate because it is derived from statistical physics. For example, if you select the parameter *m* = 2 and *r* = 0.15 in SamEn, the data points needed are 500–1000 points [18]. In MSE, the scale one is SamEn, and the data points should be sufficient to calculate SamEn. But the scale 2 is only half the data points because scale 2 is computed by taking the average of every 2 points, and scale 3 is computed by taking the average of every 3 points, and so on. In calculating the data points to scale 20, there may be insufficient data points for low sampling frequency such as 200 Hz. Because most absence seizures last <15 s, it is impossible to get enough ictal data points for analysis in low sampling frequency. In addition, short-lived transient effects may be better captured by higher sampling frequency within short data epochs. Therefore, in the present study, the scale factor was set at 1–10 for EEG data of 200 Hz and 1–20 for EEG data of 1000 Hz to get enough data for analysis. This was performed to ensure that the shortest coarse-grained time series had around 400–1000 data points.

### Complexity index

By calculating the area between the curve of MSE and the axis of scale factors, the complexity index (CI) over the time scales can be determined. CI was different from the irregularity computed by SamEn. Considering the status from all scales (frequencies), MSE is a more straightforward way of presenting the complexity of cerebral EEG signals [27, 28]. Because CI was closely related to the scale factor and larger scale factors required more data points to analyze, only EEG data with 1000 Hz sampling rate was used in this part. Therefore, a total of eight patients and 21 seizure episodes were used for analysis.

To understand the capability of CI to exhibit the brain’s circumstance, variations of CI from the pre-ictal state to the ictal state were calculated in EEG. We estimated CI using 3 s epochs, in 15 s of pre-ictal and 15 s of ictal data. The CI value was calculated from 3 s data points rather than 10 s data points to clarify the transient changes before ictal state. The 3 s period was used because a 3 s period had 3000 point data for analysis and could be calculated to scale 5. The CI changes using at least scale 5 could reach statistical significance in the ictal state in most channels. Through the CI calculations, investigations over the entire brain were more intuitively performed on each channel area by marking different CI values into different colors.

### Statistical analysis

The EEG files for analysis were available from the EEG workstation and the dataset for analysis in this study could be obtained upon request. Statistical analysis for comparison between pre-ictal and ictal states in each time scale was performed using the *t-*test and analysis of variance (ANOVA). The CI differences in different channels between pre-ictal and ictal states were compared by ANOVA, followed by post-hoc tests. Pearson correlations between different seizures in the same patient or different seizures in different patients in the ictal and pre-ictal states were calculated to investigate the relationship between different seizure episodes. Statistical significance was set at *p* < 0.05.

## Results

Twenty-one patients with 55 absence seizure episodes were included in this study. All seizure episodes showed typical clinical manifestations and 3 Hz generalized spike-waves in the EEG during seizure episodes. The demographic data, including the sex, age of seizure onset, and EEG signals are summarized in Table 1. The anti-epileptic drugs were prescribed after EEG examinations and diagnosis.

**Table 1:** Demographic data of children with absence epilepsy (n = 21). The EEG was done before anti-epileptic drug was given.

Case	Sex	Age of onset	EEG	Number of seizures	Average duration of seizures (sec)
1	F	8y	GSW, FS	3	7
2	M	5y	GSW	2	9.5
3	F	6y	GSW	2	8
4	M	6y	GSW, FS	2	8.5
5	F	5y	GSW, FS	2	8
6	F	7y	GSW, FS	4	9
7	F	7y	GSW	2	7.5
8	M	3y	GSW	2	8
9	F	6y	GSW, FS	4	9.3
10	M	9y	GSW, FS	3	8
11	M	7y	GSW, OS	2	9.5
12	F	6y	GSW, OS	2	10.5
13	F	5y	GSW	2	6.5
14	F	8y	GSW, FS	3	7
15	F	5y	GSW, OS	2	7
16	F	7y	GSW, FS	4	8.2
17	F	6y	GSW, FS, OS	3	8.5
18	M	7y	GSW, CS, PS, OS	3	7.4
19	M	9y	GSW, FS	4	9.3
20	F	8y	GSW, FS	2	7.5
21	F	12y	GSW	2	7
Total	7M/14F				
Mean		6.8±1.9y		2.6±0.8	8.2±1.1 sec

Abbreviations: M, Male; F, Female; GSW, generalized spike-waves; FS, frontal spikes; OS, occipital spikes; PS, parietal spikes

### Entropy of pre-ictal state versus ictal state

**Fig 2:**
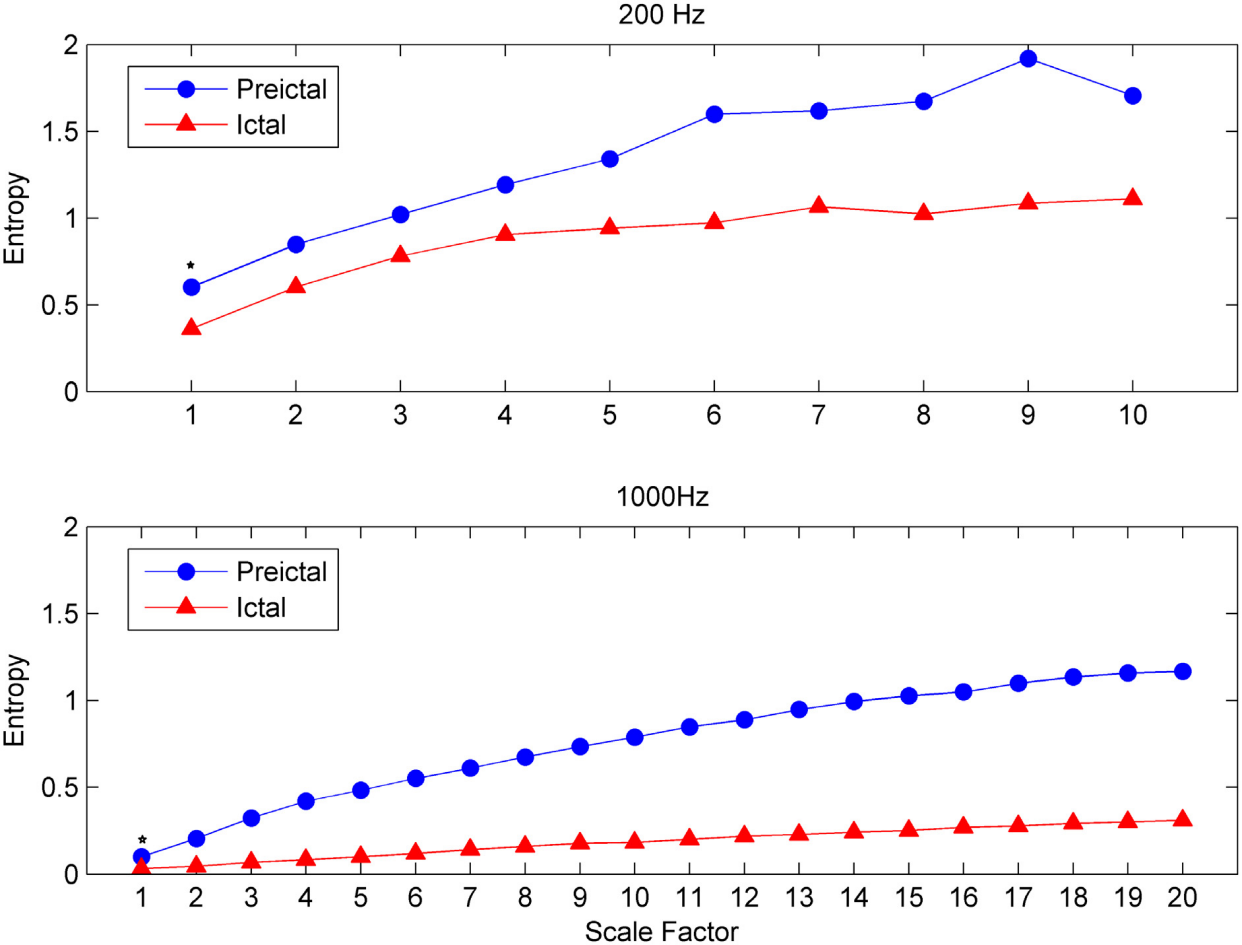
The multiscale entropy from a single seizure in a single patient. Multiscale entropy of preictal (blue line) and ictal (red line) states from a single seizure in a single patient, respectively, showing that multiscale entropy with higher sampling frequency (1000Hz, lower panel) can reveal greater difference between ictal and pre-ictal states than lower sampling frequency (200Hz, upper panel). The curve also seems smoother and can be calculated to a larger scale factor. Due to more data points in high sampling frequency, the points on high sampling frequency (lower panel) at scale 20 are corresponding to points on low sampling frequency (upper panel) at scale 4. Each panel represented MSE from one seizure in one patient. The sample entropy was the entropy at scale 1 (marking “*” in the figure).

**Fig 3:**
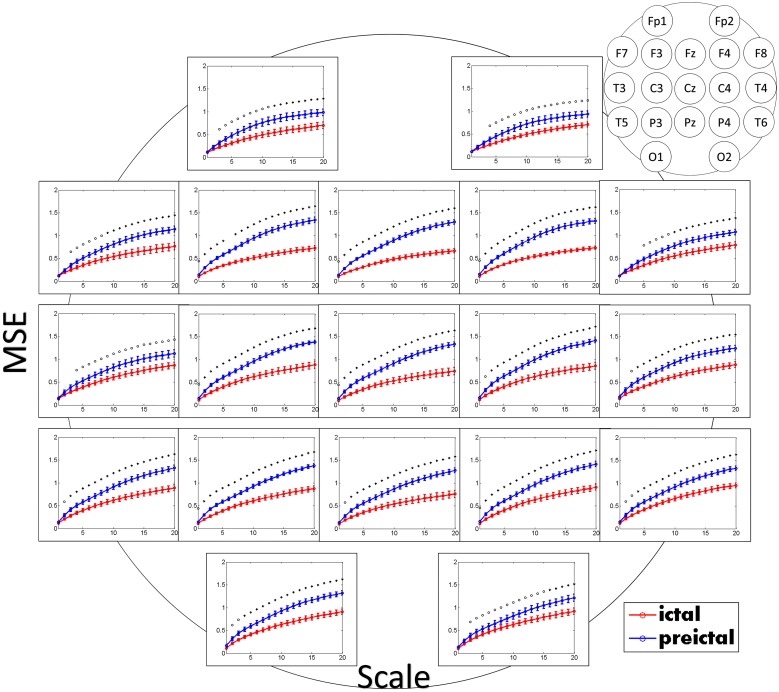
The mean of multi-scale entropy of pre-ictal and ictal states in all patients with sampling frequency of 1000 Hz. Pre-ictal state was represented by the blue line with dots and ictal state was represented by the red line with triangles. The x-axis represented the scale factor and the y-axis represented the multi-scale entropy. The error bars were standard errors. *p<0.01; ^o^ *p*<0.05.

Results from the EEG data of 1000 Hz sampling frequency revealed more detailed information and was calculated to a larger scale factor than those of 200 Hz (Fig 2). The points on 1000 Hz at scale 20 in Fig 2 were corresponding to the points on 200 Hz at scale 4. Differences in MSE values in the pre-ictal and ictal states were also significantly larger in those using 1000 Hz sampling frequency. The MSE values of 19 channels in the pre-ictal and ictal states are shown in Fig 3. It showed the decrease of MSE in all channels during ictal state. The decrease of MSE in all channels during ictal state indicated that the complexity of brain decreased and the brain remained at a homogeneous activation state in ictal state. The results showed that SamEn, the entropy in scale 1, had a relatively poor performance than MSE in detecting the difference between pre-ictal and ictal states in absence seizures (Table 2). Most channels did not reveal statistical significance in SamEn.

**Table 2:** Sample entropy in different channels in pre-ictal and ictal states, showing there were no significant differences in most channels of pre-ictal and ictal state.

	Sample Entropy (Mean±Standard error)		
	ictal	preictal	P value
Fp1	0.10±0.01	0.11±0.02	0.642
Fp2	0.11±0.00	0.11±0.02	0.872
F3	0.10±0.01	0.14±0.01	0.038
F4	0.10±0.01	0.15±0.02	0.050
Fz	0.10±0.00	0.13±0.01	0.068
F7	0.11±0.01	0.12±0.02	0.742
F8	0.12±0.01	0.12±0.02	0.993
C3	0.11±0.01	0.15±0.02	0.119
C4	0.11±0.01	0.16±0.02	0.101
Cz	0.10±0.01	0.14±0.02	0.081
T3	0.13±0.01	0.14±0.03	0.667
T4	0.13±0.01	0.18±0.03	0.285
T5	0.11±0.01	0.14±0.02	0.261
T6	0.11±0.01	0.15±0.02	0.204
P3	0.10±0.01	0.14±0.01	0.082
P4	0.11±0.01	0.15±0.02	0.090
Pz	0.10±0.01	0.13±0.02	0.138
O1	0.11±0.01	0.16±0.03	0.140
O2	0.10±0.01	0.13±0.02	0.237

In MSE analysis, the difference in MSE became larger at larger scale factors and showed no significant discrimination at smaller scale factors in channels of Fp1, Fp2, F7, F8, T3, T4, and O2. MSE in all channels revealed statistically significant changes in the pre-ictal and ictal states after scale 5. Comparing MSE changes in the inter-ictal state with age-matched control (Table 3), there was no significant change in MSE, indicating that the change in MSE came from the absence seizure. Because only new cases of absence seizures without medications were enrolled, the MSE changes were not due to the medications.

**Table 3:** The complexity index for age-matched controls and inter-ictal, pre-ictal and ictal states of absence seizures showing no difference in age-matched controls, inter-ictal, and pre-ictal state. However, there was significant decrease of CI values in ictal state compared with pre-ictal state (P < 0.05).

Channel	F3	C3	P3	F4	C4	P4	T3	T5
Inter-ictal	12.2±3.0	12.6±1.6	12.9±1.5	13.5±1.5	12.7±1.9	12.7±1.7	11.4±3.4	12.4±1.1
Pre-ictal	10.5±2.8	11.9±2.5	11.1±3.4	11.6±2.8	11.6±2.7	11.6±2.9	11.6±2.8	11.6±2.9
Ictal	6.6±2.7	8.2±2.6	8.2±3.3	5.9±1.7	7.5±2.2	8.0±2.2	6.9±2.0	7.9±2.7
Control	11.7±2.2	11.8±2.6	12.9±1.6	10.5±3.6	11.8±3.0	9.6±2.4	10.9±1.6	11.9±2.6
Channel	T4	T6	Fz	Cz	Pz	O1	O2	
Inter-ictal	11.5±1.9	12.7±1.4	11.8±1.7	11.2±2.1	11.7±1.2	12.4±1.1	11.8±1.1	
Pre-ictal	11.4±2.8	11.4±2.8	9.6±2.5	11.2±3.2	11.7±2.8	11.7±2.7	11.7±2.6	
Ictal	6.6±1.9	8.2±2.4	7.4±2.6	6.9±2.7	8.2±2.8	7.9±2.7	8.6±3.0	
Control	11.5±2.2	11.4±3.6	10.2±3.3	11.5±2.8	11.0±2.4	12.6±2.1	11.5±0.5	

### Variation of complexity index

Entropy values that were computed to multi-scale were apt to show the complexity of the brain more accurately. The CI further clarified the changes of brain network over multiple time scales. In this study, the sampling frequency also affected the CI, with higher CI in higher sampling frequency for the pre-ictal state (data not shown). This indicated that higher sampling frequency may be more sensitive to detect CI changes in the ictal state.

**Fig 4:**
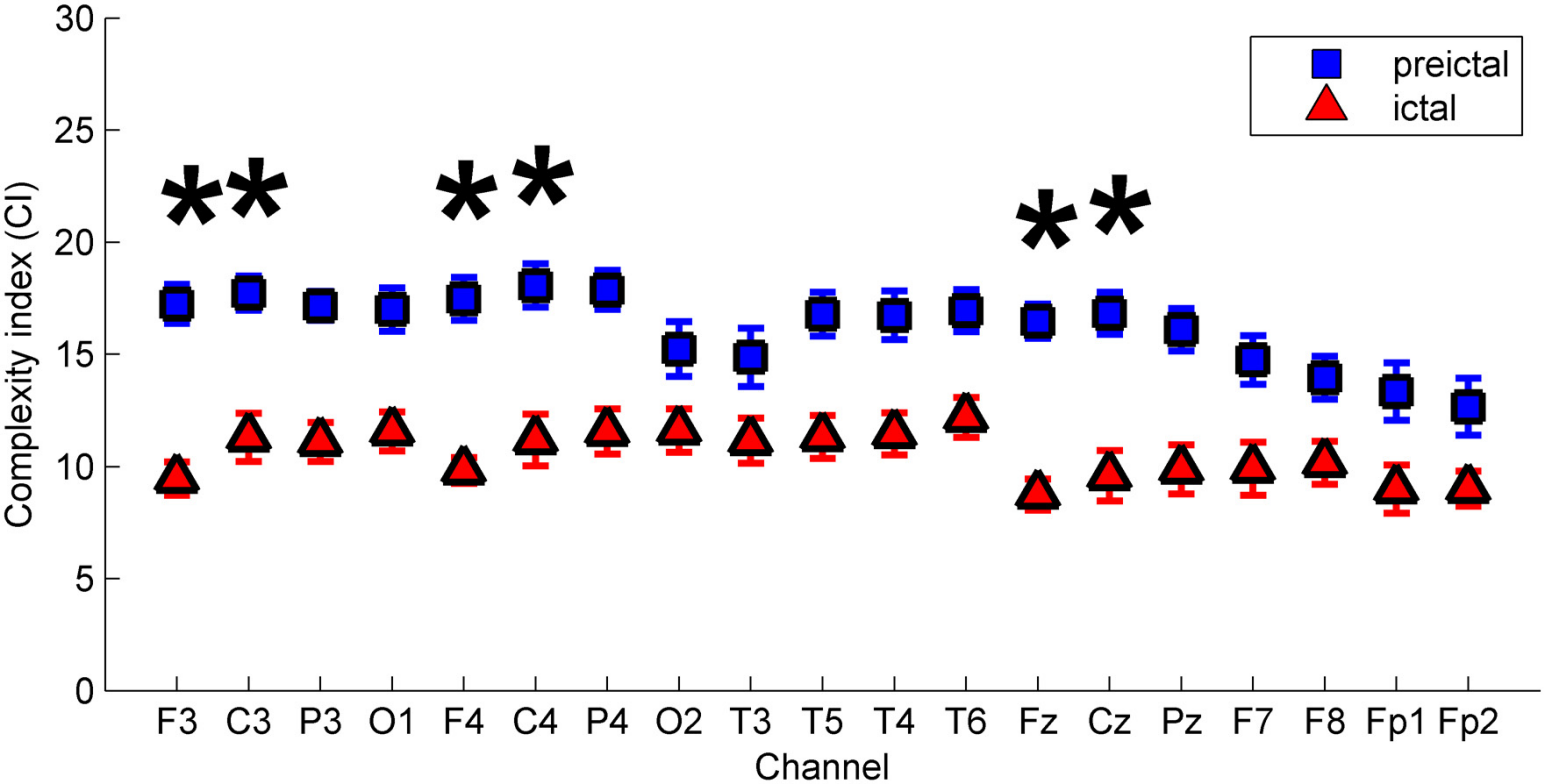
The mean of complexity index (CI) in pre-ictal and ictal states in all patients with sampling frequency of 1000Hz. Comparing CI changes in the pre-ictal and ictal states in the occipital areas, the changes were significantly larger in F3, F4, Fz, C3, C4, and Cz. The error bars were standard errors. *p<0.01.

Changes in CI values in the pre-ictal and ictal states also varied in different channels (Fig 4 and Table 3). The occurrence of absence seizures (ictal state) decreased the CI in all channels (Table 3). However, compared to changes in the occipital areas, the decrease of CI in F3, C3, F4, C4, Cz, and Fz were significantly larger during seizures (*p*<0.01). The greater decrease in CI during absence seizures in the frontal and central areas might support the fronto-central cortical involvement of “cortico-thalamo-cortical circuity” in the occurrence of generalized spike and wave discharges in absence seizures [4, 5, 7].

**Fig 5:**
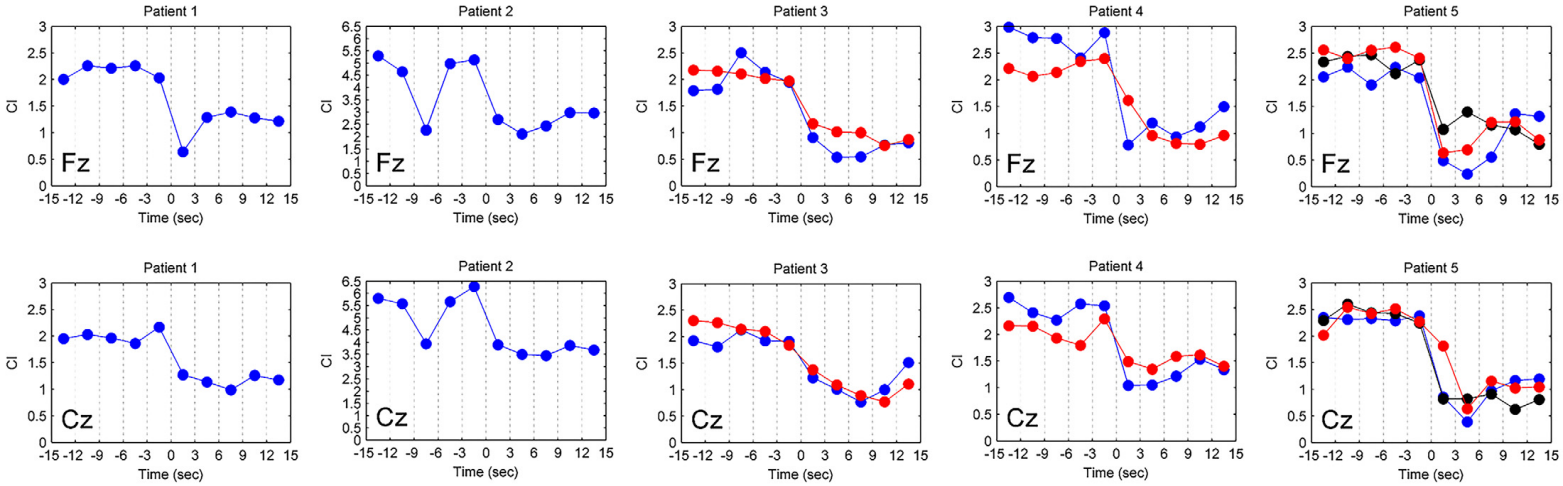
The variations of complexity index (CI) in different time period for five patients. Variations of CI at channels Fz and Cz in different time periods for five patients showed consistent CI changes in different seizures during the ictal states for the same patient. In contrast, the CI changes in different patients were not consistent. The different colors represented different seizure episodes in the same patients.

Furthermore, in order to understand the capability for exhibiting the brain’s circumstance by CI, variations of CI from the pre-ictal to the ictal states of EEG signals were analyzed, with 3 s per interval (Fig 5). The CI changes in different time periods of 5 patients from channels Fz and Cz were plotted. CI value is normally high in pre-ictal state, and decreases in ictal state as shown in the Fig 5. Using correlation approach, CI changes in the ictal state from different seizure episodes of the same patient were significantly correlated. In contrast, the correlation of CI values was poor among different patients in the ictal state and in different seizures in the same patient in the pre-ictal state (data not shown). These indicated that CI changes were consistent across different absence seizures of the same patient but were quite different from patient to patient in ictal states.

**Fig 6:**
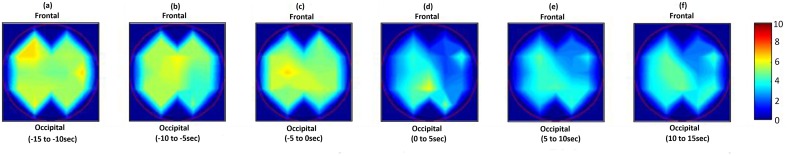
The variations of complexity index (CI) from epoch a to f in patient 1. Each epoch represented mean CI value calculated from 5 sec EEG data points from the pre-ictal state to the ictal state. Changes in mean CI were more prominent over the frontal and central areas during ictal state. The top of the figure was the front of the head and the bottom of the figure was the occipital area of the head. Blue color represented low CI value while red color represented high CI value.

In addition, even in the pre-ictal state, the correlation between different seizures in the same patient was low. In order to display variations of CI more intuitively, the changes of CI in different time periods from pre-ictal states to ictal states were plotted from Patient 1 (Fig 6). From this figure, a more intuitive way of investigating the CI variation in different time periods was attained to determine the network dysfunction of the brain. The CI values were dramatically reduced during the ictal state, especially over the frontal and central areas.

## Discussion

Recent advances in understanding the pathophysiology of absence epilepsy suggest that absence seizures may not be truly generalized seizures from the beginning, and various cortical or sub-cortical activation preceding generalized seizures have been documented [4–7, 9–11]. The present study was designed to determine the spatial and temporal connectivity of brain in absence seizures via measuring EEG complexity using MSE analysis. Our findings clearly demonstrate that the complexity of EEG signals in the ictal state are decreased, apparently mainly over the frontal and central regions, with a similar pattern in the same patient, whereas the correlation of the CI pattern in the ictal state is poor among different patients. Moreover, the data herein support our observation that EEG signals with a sampling frequency of 1000 Hz provide better resolution of serial complexity changes in a short time period from pre-ictal to ictal states.

Changes in brain biological complexity can be revealed more clearly using the MSE method rather than the irregularity calculated by the SamEn method. MSE analysis can distinguish differences between pre-ictal and ictal states more clearly at a higher scale factor. Recently, the scale-dependent Lyapunov exponent (SDLE) algorithm applied in heart rate variability has been claimed to be better than other complexity measures such as the Hurst parameter, the SamEn, and MSE [29]. Therefore, we may use SDLE algorithm to detect dynamical changes of epileptic seizures from EEG recordings in the future. Another disadvantage of the present research was that the patients were collected by sampling frequencies of both 1000 Hz and 200 Hz due to installing the newer machine recently. The points on 1000 Hz at scale 20 in Fig 2 corresponded to the points on 200 Hz at scale 4. According to sampling theory, we can only capture 500 Hz and 100 Hz signals for sampling frequency 1000 and 200 Hz, respectively. Therefore, by collecting EEG data using sampling frequency of 1000 Hz, it could get more data points for analysis and lead to a longer scales when doing MSE analysis, and clarify more detailed information of brain. If we can obtain the EEG data points in higher sampling frequency, we may also clarify more functional change of the brain. Furthermore, the scale factors in MSE analysis are also corresponding to frequency range in EEG. The MSE analysis at smaller scale factors reflect temporal dynamical complexity of higher frequency signals, while larger scale factors reflect those at lower frequencies [30, 31]. Therefore, MSE can be used to detect the frequency spectrum like low frequency waves of delta, and theta, and high frequency waves of alpha, beta, and gamma according to different scales [31]. Some diseases may significantly reduce complexity at smaller scale factors but other diseases may show abnormalities at large scale factors [30, 31]. In our case, the higher scale factors provide more difference in this population indicating that absence seizure with 3 Hz spike-waves during ictal state were driven by low frequency changes. However, this would be an interesting topic to investigate if MEG or cortical recordings can be simultaneously recorded to do the comparison in the next stage study.

As mentioned above, short-lived transient effects may be better captured by higher sampling frequency within short data epochs, and shorter data epochs are also useful when the data are irregular as in the case with EEG. Therefore, EEG signaling with sampling frequency of 1000 Hz can ensure the collection of enough data points in a shorter period. In addition, temporal changes of CI and MSE in 1–2 s, especially in the pre-ictal state, can be demonstrated more clearly using high sampling frequency, which cannot be obtained using low sampling frequency.

The analysis of complexity of the EEG signals is thought to reflect the ability of the neuronal network to adapt and function in a changing environment [24]. This study shows that entropy values and the CI at the pre-ictal state have a higher value than those at the ictal state. The shift from high-complexity towards low-complexity dynamics following transition to the ictal state provides evidence that physiologic variability or heterogeneity of brain electrical activity are lost at the ictal state. Similar results have been reported in temporal lobe epilepsy using other non-linear measures [32]. The findings in the present study are also compatible with the general hypothesis that decreased entropy or CI is associated with diseases such as epilepsy.

Through simultaneous EEG and EEG-fMRI analysis in some epileptic studies, the spatio-temporal variation of brain activity can be detected clearly during seizure episodes [14, 33]. However, through CI analysis in different EEG channels, the brain’s situation during seizures can be understood in an easier way. Previous EEG-fMRI studies show evidence of a variety of cortical or sub-cortical activations preceding generalized seizure in absence epilepsy [9–11, 14]. In rat models of absence seizures, studies have showed a “cortical focus” within the perioral region of the somatosensory cortex [34, 35]. Recent animal and magnetoencephalography (MEG) studies further demonstrated that generalized spike and wave discharges may originate from the lateral fronto-parietal cortical area [5, 7]. The results here also show that changes in CI values in the pre-ictal and ictal states are most prominent over the frontal and central areas. The CI values are dramatically reduced during the ictal state especially over the frontal area (Fig 6). These may provide the evidence of frontal involvement in absence seizures as in previous EEG-fMRI studies [10, 13]. In the present study, the significance of drop in CI in Fig 5b remained unknown. It did occur in some patients in pre-ictal state. In EEG-fMRI study [10, 13], there was activation of some brain areas before the onset of absence seizure in some patients. We suppose that there is also decrease of CI in some areas of brain before the onset of absence seizure in some patients. Because such changes are most prominent in MSE with a sampling frequency of 1000 Hz, higher sampling frequency in MSE analysis may be needed to obtain more information in investigating brain function. Measurements of MSE and CI in childhood absence epilepsy may also help clarify the role of the cortical network in the generation of generalized spike-waves in the future.

Several theories about the generation and development of absence seizures have been proposed in recent years [4]. Previous EEG-fMRI studies reveal that BOLD signal changes are consistent across different absence seizures of one patient, but are quite different from patient to patient [11]. In the present study, CI variation during different seizure episodes of the same patient revealed a similar pattern with high correlation coefficients, suggesting that the brain remains at a homogeneous activation state of similar degree in the same patient during different absence seizures. In contrast, the correlation of CI variations is poor in different patients in this study, implying clinical diversity and heterogeneity in absence patients.

In conclusion, the present study demonstrates a significant decrease of complexity during ictal state in absence seizures, mainly in the frontal and central regions. A pattern of decreased complexity is highly correlated in different absence episodes from the same patient but not from patient to patient. The results corroborate the altered brain function in children with absence epilepsy and the diverse pathophysiology of absence epilepsy. Lastly, MSE with a wide range of time scales is a useful and sensitive method for exploring brain dysfunction in absence seizures for further study.
